# Massive Retroperitoneal Hematoma After Inguinal Hernia Repair Using Prolene® Hernia System: A Case Report

**DOI:** 10.7759/cureus.43300

**Published:** 2023-08-10

**Authors:** Takahisa Fujikawa, Keiji Nagata, Taisuke Matsuoka, Ippei Yamana

**Affiliations:** 1 Surgery, Kokura Memorial Hospital, Kitakyushu, JPN

**Keywords:** prolene hernia system, postoperative complication, retroperitoneal hematoma, preperitoneal hematoma, inguinal hernioplasty

## Abstract

Retroperitoneal hematoma is a possibly fatal condition that is frequently observed as a complication of procedures such as femoral artery catheterizations. We currently present a case of massive retroperitoneal hematoma after inguinal hernioplasty using Prolene^®^ Hernia System mesh in a warfarin-treated patient. Especially in the case of inguinal hernioplasty in a patient receiving warfarin therapy, surgeons must pay close attention to prevent hemorrhage from the preperitoneal space, or they may opt for a different technique, such as the Lichtenstein method or laparoscopic approach.

## Introduction

The lifetime incidence of abdominal wall hernias is 27% for men and 3% for women [1]. The inguinal hernia is the most prevalent type in both sexes, and inguinal hernia repair is one of the most frequent operations performed in routine surgical care [2,3]. Various types of tissue repair have been documented in surgical literature since the first description of the Bassini technique in 1887 [4].

The benefits of anterior repair of inguinal hernias include minimal operative costs, a relatively brief learning curve, consistent outcomes at all levels, and the option to use local anesthesia [5]. Gilbert published the use of the Prolene^®^ Hernia System (PHS; Ethicon Inc., Somerville, NJ) bilayer patch device for treating an inguinal hernia [6]. This device is distinguished by its component attachment; its underlay patch offers a preperitoneal fix; its connector has the desirable plug characteristics; and its onlay patch encompasses the rear wall. Literature indicates that PHS repair yields superior results to Lichtenstein repair [7,8].

Despite these benefits, anterior repair with PHS might result in severe consequences not usually associated with conventional repair, such as deep infection or deep bleeding. Retroperitoneal hematoma is a possibly fatal condition frequently observed as a complication of procedures such as femoral artery catheterizations [9]. Here, we present a case of significant retroperitoneal hematoma following PHS-assisted inguinal hernioplasty in a warfarin-treated patient.

## Case presentation

A 68-year-old male presented with a left inguinal hernia. He first noticed swelling in his left groin three years ago. Recent pain in the same area that tends to worsen necessitated surgical intervention. He received long-term warfarin and aspirin therapy after an aortic valve replacement using a mechanical valve for aortic regurgitation. He had been given several hypertensive medications, and his blood pressure had been stable recently. He did not have any other chronic disease or previous history of laparotomy or bleeding events. On examination and laboratory tests, his body mass index was 28.4 kg/m^2^, and his blood pressure, hemoglobin level, and platelet count were within normal parameters. His preoperative antithrombotic management consisted of cessation of aspirin seven days before the operation, cessation of warfarin five days before the operation, and heparin bridging using unfractionated heparin (10,000 units/day).

Under general anesthesia, the left inguinal hernioplasty using the PHS mesh via an anterior approach was performed. During the operation, the spermatic cord was carefully dissected and separated, and the hernia was diagnosed as a direct inguinal hernia. After the preperitoneal cavity was manually dissected cranially and medially, a tension-free hernioplasty was completed using a large-sized PHS mesh without severe bleeding.

**Figure 1:**
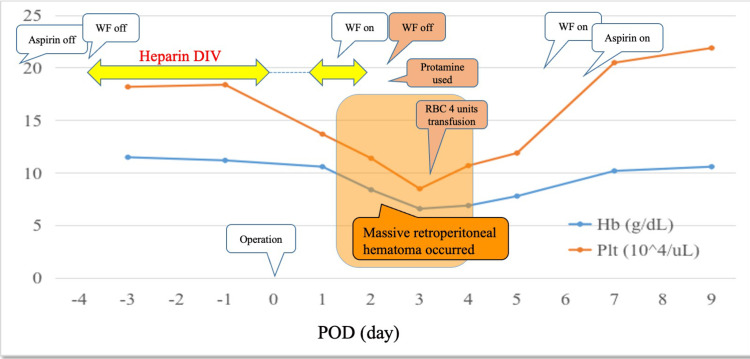
Clinical course of the current patient suffering from a massive retroperitoneal hematoma After receiving left inguinal hernioplasty using Prolene^®^ Hernia System, he suffered from left lower flank swelling and pain on postoperative day 1 and was diagnosed with a massive retroperitoneal hematoma on contrast-enhanced CT. Management was conservative, consisting of bed rest, analgesics, an infusion of protamine, and cessation of warfarin. The patient gradually recovered, and warfarin therapy was restarted without complications. DIV: Drip intravenous infusion; WF: Warfarin; RBC: Red blood cell; Hb: Hemoglobin; Plt: Platelet count; POD: Postoperative day.

**Figure 2:**
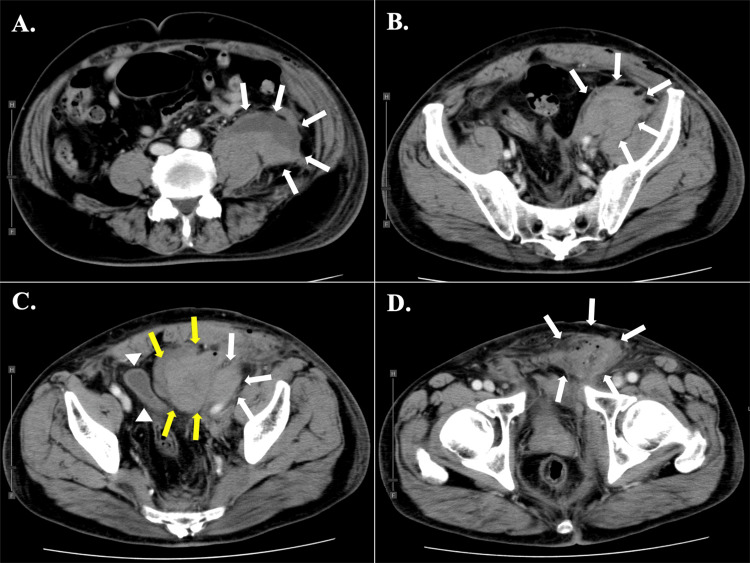
Contrast-enhanced CT images on postoperative day 2 after left inguinal hernioplasty using PHS (A, B) Massive retroperitoneal hematomas from the retropubic space to the lower retroperitoneal space were detected (white arrows). (C) The hematoma was contiguous to the preperitoneal space (yellow arrows) and severely compressed the urinary bladder (white arrowheads). (D) A contiguous left groin hematoma was also detected (white arrows). PHS: Prolene^®^ Hernia System.

Figure 1 summarizes the perioperative clinical course of the current patient. Postoperatively, the patient was well on the day of the operation. Drip intravenous infusion of unfractionated heparin (10,000 units/day) was restarted six hours after the operation, and warfarin therapy was started on postoperative day (POD) 1. The patient suffered from left lower flank swelling and pain on the night of POD1, and the symptom deteriorated on POD2. Contrast-enhanced CT of the abdomen showed massive left-sided retroperitoneal hematoma and contiguous preperitoneal hematoma, which compressed the urinary bladder (Figure 2).

Management was conservative, consisting of bed rest, analgesics, fluid resuscitation, an infusion of protamine, and cessation of warfarin. Laboratory tests on POD3 showed a low hemoglobin level (6.6 g/dL; preoperative level, 11.2 g/dL), and four units of red blood cells were transfused. Afterward, the patient gradually recovered, and warfarin therapy was restarted on POD5 without complications. He was discharged home on POD22. The patient was doing well for six months after his discharge, and no additional symptoms concerning the recurrence of retroperitoneal hematoma were seen.

## Discussion

Lichtenstein introduced the concept of tension-free repair for hernias, and mesh repairs have become widespread and are presently considered the gold standard [10]. In 1991, laparoscopic hernia repair had been developed based on the Stoppa method [11]. Following this, Gilbert reported using a bilayer patch device known as PHS to repair the inguinal hernia [6]. Literature indicates that PHS repair yields superior results to Lichtenstein repair [7,8]. In spite of these benefits, anterior repair utilizing PHS might result in relatively uncommon but severe complications not associated with conventional repair, such as deep infection or deep hemorrhage.

According to recent literature, perioperative complications occur in 8%-10% of all inguinal hernia surgeries, with bleeding (0.9%) being the most frequently observed complication [12]. The most common hemorrhagic complication following inguinal hernioplasty is groin hematoma, which is typically treated conservatively. Retroperitoneal hematoma is a well-known, but uncommon, potentially fatal clinical condition [9]. It is most commonly observed as a consequence of interventions such as femoral artery catheterizations or pelvic trauma [9]. In contrast, the massive retroperitoneal hematoma following inguinal hernioplasty, as in the current case, is an extremely uncommon but potentially fatal complication.

During inguinal hernia repair, multiple vessels, including the inferior epigastric vessels and corona mortis, may be damaged. Corona mortis is a vessel that communicates with the external iliac and obturator vessels. As a consequence of intraoperative injuries during surgical management of fractures, trauma, or hernia, it is a common source of severe bleeding [13]. During hernia repair, anatomical understanding of the corona mortis is especially crucial when performing anterior repair using PHS, which requires blind wide dissection in the preperitoneal space. The current case showed that when dissecting the retropubic area, it is important to pay attention not only to individual variations of the corona mortis but also to securing the operating field to prevent vascular injury.

Multiple sources indicate that both the status of a recurrent hernia and the use of warfarin are independent risk factors for the development of a groin hematoma following inguinal hernia repair [14,15]. Anterior approach to inguinal hernioplasty using PHS requires blind wide dissection in the preperitoneal space, which may result in potential bleeding complications. In the case of an inguinal hernioplasty in a patient receiving warfarin therapy, as in the present case, surgeons must pay special attention to prevent hemorrhage from the preperitoneal space, or they may opt for an alternative technique such as the Lichtenstein method or laparoscopic approach.

There are some limitations to the current investigation. First, as a single case, the results cannot be generalized. More research would be needed to establish the incidence of this complication with PHS repair. Second, the rationale for the appropriate form of hernia repair in a warfarinized patient is not adequately discussed due to the paucity of literature regarding the prevalence of retroperitoneal hematomas specifically after hernia surgery. Further study would be needed to definitively establish the rates of hematoma with different repair techniques and risk factors.

## Conclusions

We presented a case of massive retroperitoneal hematoma after inguinal hernioplasty using PHS in a warfarin-treated patient. Using the PHS mesh to repair an inguinal hernia necessitates extensive dissection in the preperitoneal space, which may result in potential bleeding complications. Surgeons may opt for an alternative technique, such as the Lichtenstein method or laparoscopic approach, when performing inguinal hernioplasty on patients receiving warfarin treatment.
